# Methodist Preachers

**Published:** 1862-12

**Authors:** 


### METHODIST PREACHERS.

A YEAR or two ago, a keen business-man lent "a broken-down Methodist preacher" nine hundred dollars, without any other security than his knowledge of human nature gave him. We need not stop here to eulogize the activity, the self-denial, and the indomitable energy of that somewhat contemned class, "Methodist Circuit riders," of an age agone, on that most unjust of all grounds, of there having been a few among thousands (fewer in proportion, by far, than in the Saviour's little company of twelve), who were unfaithful to their high trusts. But where one was unfaithful, there were scores, and hundreds, and, perhaps, thousands, who lived long, labored hard, and died working—died in their devotion—died with their armour on—died in penury, and went down to unrequited, unhonored, and forgotten graves; unrequited, unhonored, and forgotten, as to man, but remembered in Paradise by the Master, to be honored at the Judgment by the Maker of all Worlds, when conquerors and kings will be passed without a single recognition.

Not a few, after having worn themselves out in the service of the Church, after having become disabled by disease, find themselves turned into the great field of the world, to earn a living in a manner the best they may. The subject of this narrative was one of these; and, carrying with him that independence and energy and willingness to the exercise of that self-denial which he had full large opportunities of cultivating in his humble calling of "circuit rider," he determined that he would go to work in a mode nearest akin to his legitimate calling, and most likely to renovate his health, as well as to replenish his purse. He went to a friend—and those who are neither afraid nor ashamed to do any lawful thing, so that they can make a living thereby, seldom lack friends in need—and, with the frankness which unfaltering self-reliance only can give, said, "I want to borrow some money," and proceeded to build his castle, and a most aerial one it seemed to be at first glance. "I want to purchase a stock of twenty-dollar Bibles, to sell to poor people." A poor preacher borrowing money to purchase costly Bibles to sell to poor people, appeared to be rather a poor inducement for a business-man to lend money, without the inevitable "bond and mortgage." But a further development of the programme gave some plausibility to the plan. The money was loaned, and the Bibles purchased at the lowest publisher's cash rates. To make a beginning, and thus put him in funds for extending his operations, he took a fine Bible under his arm, made his way to Fulton Market, and promenaded its oderiferous aisles until he found a purchaser; he then returned to get another. In the course of a few weeks, in the furtherance of his original plan, he was traveling around on foot among the farmers, especially seeking out those recently married. Not one in fifty would think of giving twenty, and twenty-five, and thirty dollars for a Bible. Still, they were exhibited; they were so beautifully got up; and then again the thought would occur, "If we have anything good about the house, it ought to be the Bible;" and the mind would run back to other years, of father and mother and home, and "the dear blessed Bible that lay on the stand," ready to be opened for the morning and the evening sacrifice; and on the mind would run, following the father and mother, long since gone up higher, with the reading of the Bible as a means of re-joining them in Heaven, when life's labors were all over. But just here hard facts would come in. "We can't spare the money; there is not that much in the house." "But, perhaps, you can pay a little now, and you may lay by a dollar every week, and I will call for it." And the Bible was taken. There is no danger, thought our hero, in trusting under the circumstances. People who have a heart to purchase a family Bible, are not likely to lack the principle of honorable payment. To shorten the story, this gentleman, in less than two years, has extended his business so much, that, having recovered his health, he finds his time fully employed during the week, in the city, in keeping a good stock on hand, for his agents, who are scattered in every direction—he, a master *colporteur*, and they his *employés*, working for him. On the Sabbath he preaches as of old, and thus, in a two-fold way, is he employed in sowing the seed of the Word, supplying families with the Bible at his own cost, and with a liberal profit to himself.

With the same personal independence and self-denial and energy, who shall deny that many a minister might be restored to his legitimate calling, when, if he had lounged about Summer re-sorts and watering-places, or had "gone to Europe," or had "given up his charge," waiting for a return of health, he would have continued an invalid, infirmities growing upon him, to be ended only in the grave.

Reader! there are multitudes who are mere cumberers of the ground, between whom and extensive usefulness, there is nothing intervening, but the want of humility; they have not the moral heroism to be willing to do anything useful, to be anything, rather than do nothing, waiting for better things to come to them. Are you one of these?—

